# Supplementary material for: Metabolic Syndrome Ameliorated by 4-Methylesculetin by Reducing Hepatic Lipid Accumulation
Source: Int J Mol Sci. 2022 Sep 9;23(18):10465. doi: 10.3390/ijms231810465 (PMC9499566; doi:10.3390/ijms231810465)
Supplement: Supplementary file 1 [file ijms-23-10465-s001.zip › ijms-1886048-supplementary.pdf]

Table S1. The ingredients of high-fat diet and standard chow diet

Constituent	% (High-fat diet)	% (Chow diet)
Protein	11%	11%
Fat (Crude fat, Lard)	25%	6%
Carbohydrate<br>(corn Starch, maltodextrin, sucrose and fiber)	49%	74%
Total	91%	91%
**Ingredient**	**g/Kg (High-fat diet)**	**g/Kg (Chow diet)**
Crude protein (⩾80% protein)	140	140
Crude fat (85% fat)	120	70
Lard	150	-
Corn Starch	150	300
Maltodextrin	200	400
Sucrose	150	-
Fiber	50	50
Mineral mix (GB 14924.3-2010)	30	30
Vitamin mix (GB 14924.3-2010)	10	10
L-Cystine	3.3	3.3
Choline-bitartrate (44.4% Choline)	2.8	2.8
Total	1006.1g	1006.1g

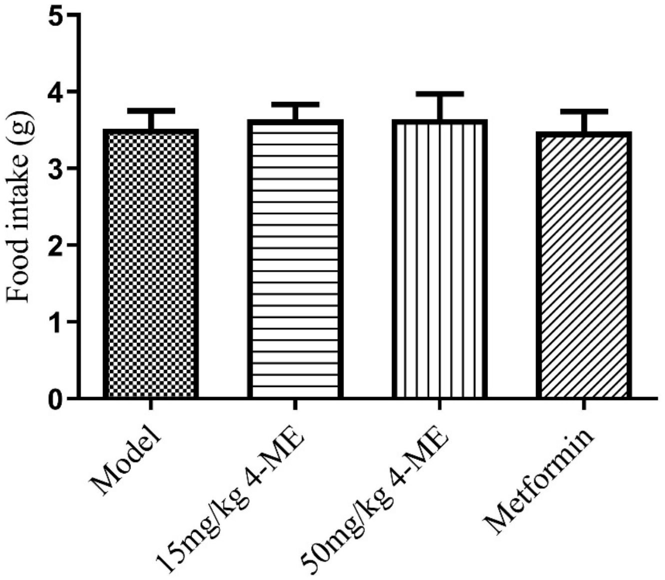

Figure S1. The effects of 4-ME and metformin upon food intake in mice. The results were expressed as mean ± SEM (n=8).

SAMPLE INFORMATION

Sample Name:	PRF8010521	Acquired By:	System
Sample Type:	Unknown	Sample Set Name:	
Vial:	9	Acq. Method Set:	6 7 Dihydroxy 4 methylcoumarin
Injection #:	1	Processing Method:	Samples
Injection Volume:	5.00 ul	Channel Name:	340.0nm
Run Time:	20.0 Minutes	Proc. Chnl. Descr.:	PDA 340.0 nm
Date Acquired:	2017-1-5 9:55:44 CST		
Date Processed:	2017-1-5 10:20:32 CST		

Auto-Scaled Chromatogram

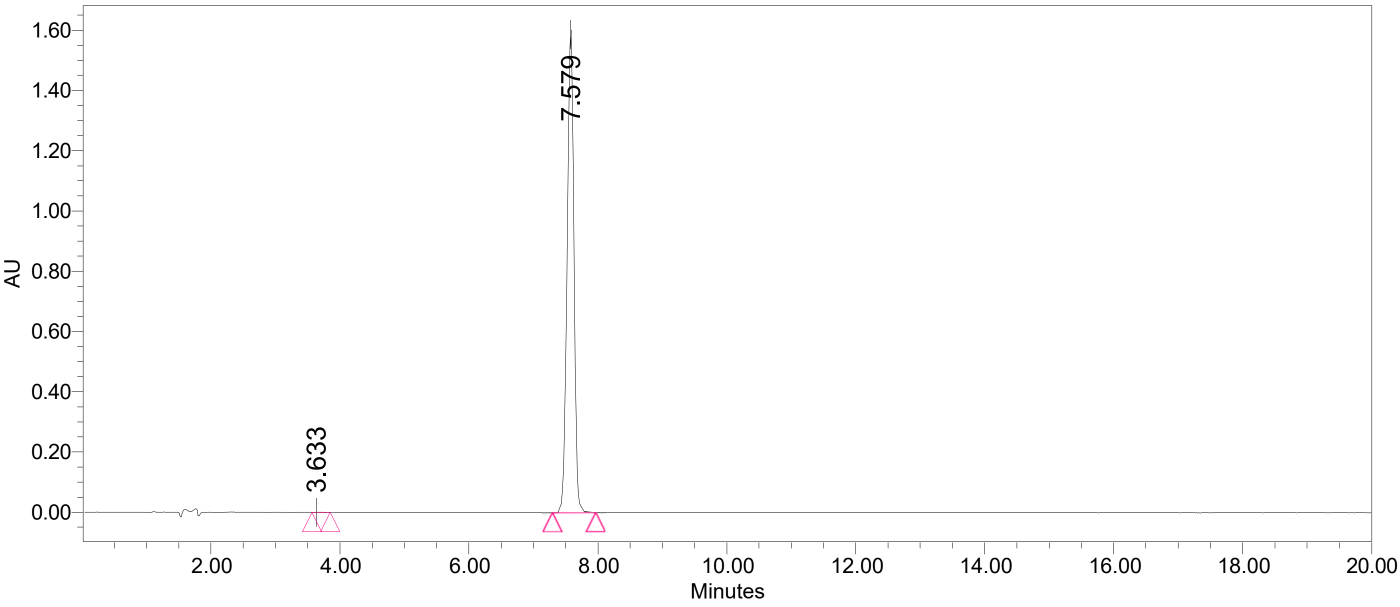

Peak Results

Peak	RT	Area	% Area	USP Plate Count	USP Resolution
1	3.633	6065	0.05	7450.8	
2	7.579	11640471	99.95	23987.6	19.7

Figure S2. HPLC of 4-Methyl-6,7-dihydroxycoumarin. Peak 2 is 4-Methyl-6,7-dihydroxycoumarin.
